# Developing a Clinically Practical Biomaterial Platform for Endogenous Liver Regeneration

**DOI:** 10.3390/gels12050426

**Published:** 2026-05-13

**Authors:** Carter Beamish, Faraz Abounorinejad, David Kim, Ai Phuong Tong, Harika Barri, Chris Marx, Daniel Lane, Hugh McGregor, Grace Laidlaw, James Jeffries, Ray Yeung, Bruce Hinds, Miqin Zhang, Ryan L. McCarthy, Kelly Stevens, Avik Som

**Affiliations:** 1 Department of Material Science and Engineering, University of Washington, Seattle, WA 98195, USA; 2 Division of Interventional Radiology, Department of Radiology, University of Washington, Seattle, WA 98195, USA; 3 Division of Gastroenterology and Hepatology, Department of Pediatrics, University of Washington, Seattle, WA 98195, USA; 4 Center for Developmental Biology and Regenerative Medicine, Seattle Children’s Research Institute, Seattle, WA 98105, USA; 5 Department of BioEngineering, Institute of Stem Cell Regeneration and Medicine University of Washington, Seattle, WA 98195, USA

**Keywords:** liver regeneration, biomaterials, tissue engineering, drug delivery, hydrogels, metallic nanoparticles, tissue scaffolds, controlled release

## Abstract

Chronic liver disease remains a major global health burden, with liver transplantation as the only definitive therapy despite severe limitations in donor availability, surgical morbidity, and patient eligibility. Although the liver has substantial intrinsic regenerative capacity, endogenous repair is often insufficient in chronic injury, cirrhosis, and acute-on-chronic liver failure. As a result, regenerative strategies that restore liver function without whole-organ replacement are increasingly pursued. This review examines controlled release biomaterial-based liver regeneration platforms, particularly those that utilize hydrogels and/or complementary nanoparticle systems, as clinically practical tools to enhance endogenous regeneration. We include discussion of both 3D scaffold-based and injectable hydrogels to enhance regeneration. Used as biological support and controlled release mixtures, they enable local retention, entrapping and controlling the release of regenerative cues including growth factors (HGF, EGF, etc.), nucleic acids for gene expression, stem cells or other cell populations, and conditioned extracellular vesicles, overcoming poor cell engraftment, short cytokine half-lives, and other limitations. Further, synthetic nanoparticles can structure release at the protein/molecular level as well as catalytically modulating oxidative stress and inflammation. Within the context of these systems, we structure the anatomical, engineering, and imaging considerations essential for the clinical translation of gel composite systems while highlighting remaining barriers to wider clinical adoption. Collectively, these advances position biomaterial-enabled regenerative therapies as a realistic alternative or bridge to donor restricted liver transplantation.

## 1. Introduction

Liver failure remains one of the most challenging clinical conditions to manage, owing to its high mortality, limited therapeutic options, and the complex biology underlying hepatic repair. Chronic liver diseases, including viral hepatitis, metabolic dysfunction-associated steatotic liver disease (MASLD), and alcohol-related liver disease, remain major contributors to the approximately two million yearly deaths from liver disease worldwide [1]. Acute-on-chronic liver failure is especially fatal, with a 28-day mortality exceeding 25% and a 90-day mortality rate around 50% even with sustained medical care, as survival often hinges on rapid access to donor transplantation (n = 303) [2]. Chronic liver disease is also associated with a significant economic burden for patients, costing an average of $3032 per month out of pocket which surpasses monthly expenditures for patients with heart failure ($2491.60) and COPD ($1955.60) [3]. Other hidden expenses include patients experiencing high rates of unemployment due to disease-related disability, and the time and financial costs transferred to informal caregivers [4].

Although liver transplantation has transformed outcomes for select patients, its impact is fundamentally constrained by donor shortages, procedural morbidity, and restricted patient eligibility [5]. Life-limiting comorbidities, including portal hypertension with associated ascites, hepatic hydrothorax, GI hemorrhage from variceal bleeding, synthetic dysfunction, coagulopathy, and hepatic encephalopathy associated with progressive liver failure may preclude patients from tolerating liver transplantation. Patients must also meet stringent transplant eligibility criteria. Though it varies based on transplant center, this often includes 6 months of alcohol abstinence, proof of adequate social support, and age-out exclusions, with the data justifying these limits remaining controversial [6]. As a result, there is growing recognition that transplantation alone cannot address the rising global burden of liver disease, motivating the search for alternative strategies that restore liver function without whole-organ replacement [1,5].

Unlike most solid human organs, the liver possesses a remarkable intrinsic capacity for regeneration. This regenerative response is orchestrated through tightly regulated interactions among inflammatory mediators, growth factors, metabolic signals, and biomechanical cues [7]. Aside from severe cases, these pathways can successfully restore liver mass and function in acute injury. However, in chronic disease states such as cirrhosis and acute-on-chronic liver failure, regenerative signaling becomes dysregulated or is insufficient; the balance between regeneration and fibrosis being progressively lost [2,7]. The resulting impairment of endogenous repair ultimately drives progressive organ failure despite the presence of regenerative stimuli.

While pharmacologic and cell-based therapies have shown promise in preclinical and early clinical liver regeneration studies, further translation has been hindered by fundamental challenges including poor localization/short biological half-lives of regenerative factors, limited cell engraftment, and concerns regarding oncogenic risk and effects on off-target tissues [8,9]. These limitations underscore the need for delivery strategies that can precisely control where, when, and how regenerative signals are presented within the diseased liver.

Biomaterial-based approaches have emerged as a powerful strategy to overcome these barriers by enabling spatial and temporal control over regenerative cues and cellular microenvironments [10]. Injectable hydrogels offer a flexible platform for localized delivery of growth factors, nucleic acids, and cells within the liver parenchyma, vasculature, or peritoneal space while minimizing systemic exposure [11]. These materials can be engineered to mimic extracellular matrix properties, protect labile therapeutics, and support transplanted cells, making them well-suited for regenerative applications [11]. Importantly, their injectability and tunable physical properties allow direct integration with image-guided, minimally invasive procedures that are already routine in clinical practice [11].

Hydrogels used for localized biomolecule delivery are chemically diverse polymer networks whose function is dictated by their molecular structure and functional group chemistry [11]. Natural polymers such as chitosan, alginate, and collagen present primary amine, carboxyl, and hydroxyl groups that mediate cell recognition, adhesion, and electrostatic interactions with encapsulated growth factors or nucleic acids [12]. These functional groups also confer environmental responsiveness, particularly to pH, allowing hydrogels to swell, degrade, or alter permeability in response to inflammatory conditions or physiological pH characteristic of liver injury [13]. Such chemically driven changes in network architecture directly regulate mesh size, biomolecule retention, and controlled release kinetics, making hydrogel chemistry central to spatially and temporally precise regenerative therapy [11].

In parallel, inorganic and metallic nanoparticles have attracted increasing interest due to their preferential hepatic accumulation and multifunctional capabilities [14]. Able to act as multifunctional catalytic antioxidants, imaging agents, and targeted delivery vehicles, these nanomaterials can modulate inflammation and fibrosis—key barriers to effective liver regeneration [14]. When combined with hydrogel-mediated local retention, nanoparticles enable hybrid systems that couple mechanical support with biochemical, catalytic, and imaging functions, further expanding the design space for localized and multifunctional regenerative therapies.

This review synthesizes recent advances in biomaterial-enabled strategies for endogenous liver regeneration, with an emphasis on clinical practicality and translational relevance. Key molecular targets of regeneration are examined alongside material systems for controlled delivery of regenerative agents, hydrogel-assisted cell therapies, tissue engineering approaches, and nanoparticle-mediated modulation of the hepatic microenvironment. Throughout the review, anatomical, engineering, and image-guided delivery considerations that govern clinical feasibility are highlighted. By clearly distinguishing regenerative concepts from existing supportive and replacement therapies, this review provides a translational roadmap for biomaterial-based liver regeneration that extends beyond experimental promise toward meaningful clinical impact.

## 2. Prospective Alternative Options to Liver Transplantation

**Figure 1:**
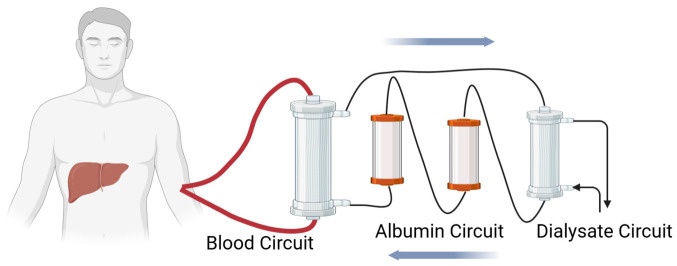
Schematic representation of the MARS for liver dialysis, a bridging therapy that can be used to keep patients alive while awaiting a donor organ adapted from Mitzner 2011 [25]. Created in BioRender. Som, A. (2026) https://BioRender.com/984o85x.

No approved therapies fully replace liver transplantation for end-stage liver failure, though several are under preclinical investigation. Extracorporeal liver support systems, such as liver dialysis approaches like the Molecular Adsorbent Recirculating System (MARS), have shown some promise (Figure 1) [15]. However, liver dialysis systems tend to be less effective than renal dialysis, as toxic metabolites associated with liver failure are albumin-bound and not as easily removed by semipermeable membrane exchange alone. Systematic reviews and meta-analyses for MARS have demonstrated improved survival for those with acute liver failure, with a risk ratio of 0.7 in one meta-analysis and 0.61 in another, but they have been unable to demonstrate benefits for those with acute-on-chronic or post-hepatectomy liver failure preventing the full clinical adoption of this treatment [15,16,17].

While the MARS is the only extracorporeal liver system approved by the FDA, other systems seek to improve its methods by incorporating additional avenues for treatment. Eleven randomized controlled trials have evaluated the Double Plasma Molecular Absorption System (DPMAS) combined with albumin and coagulation factor supplementation via plasma exchange (PE) for acute-on-chronic liver failure (ACLF), suggesting improved outcomes with combination therapy [18]. Alternatively, the Artificial Multi-Organ Replacement (AMOR) system seeks to treat multi-organ failure in liver disease by combining albumin dialysis with traditional dialysis to remove excess fluid [19]. Though its effects have yet to be evaluated in a randomized control trial, a trial of its usage on 10 patients with multi-organ failure indicated clinical improvement in all 10 patients and sustained 5 of them until transplant receipt or recovery [19].

Xenotransplantation has recently advanced, initially with humanized pig livers transplanted into brain-dead patients that demonstrated functional activity and regeneration for up to 10 days [20]. Earlier this year, this treatment was used for the first time to sustain a patient until they were able to receive a human liver transplant [21]. The clinical application of this approach remains experimental, and it also still requires the operation of transplantation and contains the same risks with treatment.

Finally, grafts of cultured primary human hepatocytes have been in development since 1992. Around 150 patients worldwide have received hepatocyte transplants since then, with methods typically involving direct injection of cells into the portal vein or splenic pulp without support [22]. Positive outcomes were limited by low engraftment rates (typically only 0.1–0.3% of the host liver mass following infusion of 3–5% of liver-equivalent cells) and immunogenicity concerns, restricting their role mainly to bridging therapies rather than long-term alternatives [22,23,24].

## 3. Mechanism and Molecular Targets for Liver Regeneration

The liver possesses the highest regenerative capacity of any organ in the human body. Under wound or stress conditions, it is capable of responding by promoting cell renewal to maintain homeostasis, a process carefully controlled through both juxtacrine and paracrine signaling. The impairment of these signals and the cellular response to them, either through interference from inflammatory cytokines or reactive oxygen species, is the hallmark of chronic liver diseases and disrupted signaling which leads to ECM overproduction causing fibrosis, pathological neo-angiogenesis that detours blood flow, loss of liver zonation, and hepatocyte dedifferentiation causing hepatocellular function loss [26,27]. The recovery of these pathways represents a promising objective for regenerative liver therapy.

The primary extracellular signals that act directly on hepatocyte regeneration are listed in Table 1. Mechanistically, studies in mice and rats indicate the signals from Kupffer cells, primarily TNFα and IL6, prime hepatocyte cell division entry from the G0 to G1 phases, while factors such as EGF, HGF, and TGFα drive progression through the remaining phases of cell division [28]. The potential of these signals to trigger hepatocyte proliferation means they can all be implicated in liver carcinogenesis (Table 1). Additionally, the maladaptive use of these signals is implicated in the progression of liver fibrosis, except in the case of HGF, TGFα, and GRP78, which have demonstrated antifibrotic effects in mice [29,30,31,32].

A necessary step in triggering hepatocyte cell division is priming from the IL6 and TNFα inflammatory cytokines. For human hepatocytes in the presence of growth factors, IL6 strongly boosts cell growth, but its use in vitro has been noted to lead to lipid accumulation without other metabolic factors to modulate its effect [33]. Another member of the IL6 family, Oncostatin M (OSM), similarly fulfills this role and additionally counters metaplasia to the biliary duct lineage [34,35]. While IL6 and OSM primarily convey their effects through JAK-STAT activation, TNFα instead activates NF-κB. Because of this difference, some in vitro studies indicate that TNFα is a more potent priming agent than IL6 in both HepG2 cells and primary human hepatocytes (PHH) [36,37].

Following hepatocyte priming, growth factors are necessary to proceed through the remaining steps in cell division. HGF is a strongly mitogenic secreted signaling factor and is the most commonly used factor in liver regeneration scaffolds. While potent, this factor has a biological half-life below 5 min in rats, primarily due to rapid hepatocellular uptake [8]. EGFR ligands possess a similar mitogenic capacity, and despite sharing a receptor, they have differing effects potentially due to other off-target ErbB receptors that are activated [38]. While some EGFR ligands such as neuregulin and epigen are not necessary for liver regeneration and have not been extensively studied (excluded from Table 1), preliminary data suggests they may have mitogenic effects and are upregulated in liver cancer [39,40]. The Wnt/β-catenin pathway induces hyperplasia similarly to HGF/EGF in human hepatocytes but uniquely triggers no such response in murine hepatocytes [37]. This marks a key difference between studies in mice and humans.

**Figure 2:**
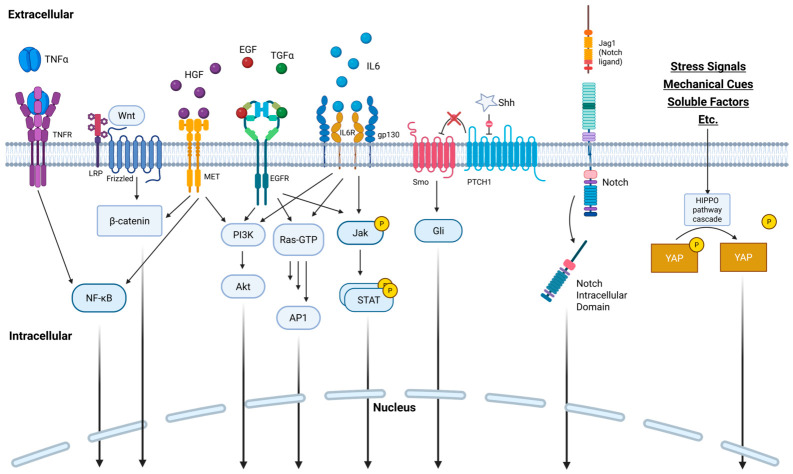
Schematic overview of the major signaling pathways upregulating hepatocyte growth and cell cycle progression during liver regeneration, as evidenced by studies in mice and rats. The diagram illustrates key extracellular cues and their downstream intracellular pathways that coordinate hepatocyte priming, proliferation, and survival following liver injury. Created in BioRender. Som, A. (2025) https://BioRender.com/aja5gtu.

These signals trigger several intracellular pathways, also listed in Table 1, to promote hepatocyte proliferation. These cascades ultimately converge on transcription factors that drive cell cycle progression, as is summarized in Figure 2 [28,41,42]. Many signals share the same cellular machinery, and there is significant crosstalk among these pathways that allows them to modulate one another’s activity and balance regenerative and fibrotic responses. For example, STAT3 activation via IL6 or HGF inhibits apoptosis, and the β-catenin pathway triggered by Wnt and HGF decreases activity of Hippo kinases which allows YAP translocation to the nucleus [35,39,43]. While most approaches involve supplementing the extracellular signals necessary to trigger these pathways, an interesting approach to treatment is in the case of an MKK4 inhibitor in the Ras pathway, which selectively inhibits certain regenerative pathways to divert signals towards pathways that better promote proliferation [44].

**Table 1:** Regenerative signals that act directly on hepatocytes. All listed factors can be implicated in the development of cancer but have different effects on the development of fibrosis. TNFα = tumor necrosis factor alpha, IL6 = interleukin 6, HGF = hepatocyte growth factor, Shh = Sonic Hedgehog protein, EGF = epidermal growth factor, TGFα = transforming growth factor alpha, GRP78 = glucose-regulated protein 78, Wnt = wingless-related integration site, JAK = Janus kinase, STAT = signal transducer and activator of transcription, PI3K = phosphatidylinositol 3-kinase, Akt = protein kinase B, PTCH1 = protein patched homolog 1, Smo = smoothened, PHH = primary human hepatocyte, HepG2 = human hepatoma G2, HIBEpiC = human intrahepatic biliary epithelial cell.

Extracellular Signals	Biological Function	Regenerative Pathway	Natural Trigger for Excretion	Primary Secreting Cells	Intracellular Pathways Triggered	Fibrotic?	Subjects Used in Cited Study	Sources
EGF	EGFR Ligand. Deletion results in poor liver regeneration.	EGFR Pathway	Liver injury.	Activated Hepatocytes, Cholangiocytes	JAK-STAT, Ras, PI3K-Akt	Profibrotic	Rats, Mice, PHH	[35,40,45,46]
TGFα	EGFR Ligand. Deletion results in poor liver regeneration following partial hepatectomy in cirrhosis but not in previously healthy livers.	EGFR Pathway	Liver injury and hepatocyte activation.	Activated Hepatocytes	JAK-STAT, Ras, PI3K-Akt	Antifibrotic	Rats, Mice, PHH	[32,40,47,48,49]
Amphiregulin	EGFR Ligand. Deletion results in poor liver regeneration.	EGFR Pathway	Liver injury, via IL-1β and PGE2.	T Regulatory Cells	JAK-STAT, Ras, PI3K-Akt	Profibrotic	Mice	[50,51,52]
Heparin-Bound EGF	EGFR Ligand. Deletion results in poor liver regeneration.	EGFR Pathway	Liver injury. Oxidative stress signals induce gene expression.	Liver Sinusoidal Endothelial Cells	JAK-STAT, Ras, PI3K-Akt	Deletion and overexpression both increase fibrosis	Mice, Rats	[40,53]
Epiregulin	EGFR Ligand. Deletion does not affect liver regeneration.	EGFR Pathway	Liver injury.	Hepatic Stellate Cells	JAK-STAT, Ras, PI3K-Akt	Profibrotic	Mice	[38,54,55]
Betacellulin	EGFR Ligand. Deletion does not affect liver regeneration.	EGFR Pathway	Liver injury.	Cholangiocytes	JAK-STAT, Ras, PI3K-Akt	Profibrotic	Mice	[40,56]
GRP78	A protein traditionally known as a major endoplasmic reticulum chaperone. Stimulated release promotes hepatocyte proliferation and leads to anti-apoptotic effects	GRP78 Pathway	Release of M2BPGi from hepatic stellate cells.	Kuppfer Cells	Presumably enacting its role as a molecular chaperone	Antifibrotic	Mice, PHH	[30]
Shh	Major signaling protein in embryonic development. Plays a role in adult liver regeneration.	Hedgehog Pathway	Cellular damage, platelet-derived growth factor, EGF, and TGFb.	Hepatic Stellate Cells	PTCH1/Smo	Profibrotic	Mice	[57,58]
HGF	Key signaling molecule in hepatocyte growth and proliferation. In healthy liver it is bound to the ECM in its inactive form (pro-HGF) but can also be produced by resident liver cells other than hepatocytes.	HGF Pathway	Urokinase-type Plasminogen Activator (uPA) is activated by increased hemodynamic stress and activates pro-HGF. Cellular HGF production is activated by pro-inflammatory cytokines (IL6 and TNFα).	Hepatic Stellate Cells, Endothelial Cells, Kuppfer Cells	NF- κB, PI3K-Akt, β-Catenin	Antifibrotic	Mice, Rats, PHH, HIBEpiC	[30,31,35,43,59,60,61,62,63,64]
Mechanical Cues/Stress Signals	Signaling pathway with various triggers that lead to Hippo pathway inhibition. The Hippo pathway typically inhibits YAP, which activates transcription and cell proliferation.	Hippo–YAP Pathway	N/A	N/A	Hippo–YAP	Profibrotic	Mice	[41,65,66,67]
IL6	Systemic pro-inflammatory cytokine that also plays a role in liver regeneration.	IL6 Pathway	Autocrine TNFα signaling.	Kuppfer Cells	JAK-STAT, Ras, PI3K-Akt	Profibrotic	Mice, PHH, HepG2	[30,36,37,57,68]
Oncostatin M	Cytokine in IL6 family. In addition to priming hepatocytes for cell division, prevents metaplasia to biliary duct lineage.	IL6 Pathway	IL6 is implicated in its release	Kuppfer Cells	JAK-STAT	Profibrotic	Mice, PHH	[34,35,69,70,71]
Jag1 (Notch Ligand)	Surface protein that is expressed to a greater extent following liver injury. Triggers Notch receptors on neighboring cells.	Notch Pathway	Liver injury leads to greater surface expression of Jag1.	Hepatocytes	Notch Signaling Pathway	Profibrotic	Rats	[42,72,73]
TNFα	Systemic inducer of inflammation for the innate immune system.	TNFα Pathway	C3 and C5 complement components. Toll-like receptor 4.	Kuppfer Cells	NF- κB	Profibrotic	Mice, PHH, HepG2	[36,37,57,74]
Wnt	The canonical Wnt pathway involves its signaling through β-catenin, which can lead to fibrosis. The non-canonical Wnt pathways either promote cell differentiation or inflammation. Important in regulating liver size and zonation.	Wnt Pathways	Liver injury.	Kuppfer Cells and Liver Sinusoidal Endothelial Cells	Canonical: β-Catenin; Non-Canonical: Rho/Rac or Ca^2+^ increase	Profibrotic	Mice, Rats, PHH	[37,75,76,77]

Some transcription products, such as non-coding RNA molecules, do not translate into functional proteins but still play a vital role in hepatocyte regeneration [78]. Among these, microRNAs (miRNAs) are rapidly up- or down-regulated to facilitate transitions through the cell cycle [79], while long non-coding RNAs (lncRNAs) can interact directly with proteins and other RNA molecules to support cell growth and division [80]. Hi-throughput sequencing technology has revealed 167 miRNAs that are differentially expressed during liver regeneration, of which ~20 have previously been shown to be differentially regulated during liver regeneration [81]. Primarily, the ones known to enhance liver regeneration include miR-21, miR-19a, miR-214, miR-203, and miR-27a/b, and they typically act through the repression of oncosupressive genes [82]. In contrast to this, over 1000 lncRNAs have been found to be differentially expressed during liver regeneration, with very few of them having a known function from previous studies [78].

Aside from the factors necessary for initiating cell division, other steps are necessary to ensure proper regeneration. One is the inhibition of TGFβ, an anti-proliferative and fibrogenic cytokine, which has been found to act via autocrine signaling in rats [83]. Its inhibition is also necessary for human hepatocyte cultures [37]. Although they do not play a role in triggering hepatocytes to proceed through the cell cycle, extracellular matrix components are key to liver regeneration by allowing cell adherence and regrowth into damaged tissue regions in addition to regulating cellular signaling [84]. Additionally, studies indicate that continuous HGF exposure in vitro leads to hepatocyte dedifferentiation in the absence of ECM components, meaning their presence is necessary for determining cell fate [85,86].

## 4. Hydrogel Biomaterial Characteristics Governing Liver Regenerative Applications

With the wide range of possible signaling methods to enhance liver regeneration detailed above, the successful regulation of that potential requires supporting materials, standardly hydrogels, and their ability to enhance the liver’s natural regenerative pathways. Beyond serving as passive carriers of secreted or adherent moieties, the gel’s biomaterial base must integrate with non-hepatic tissue, interact with local cells, and bio-integrate or degrade in a controlled manner under physiological conditions. Key design parameters across all regenerative hydrogels depend on molecular control of components and their modification levels, network structure, degradation behavior, off-target biochemical signaling, and compatibility with clinically relevant delivery routes [11,87].

The basis of any hydrogel is the basal materials used for scaffold formation, selected for their well-defined synthesis control and biocompatibility. An overview of some of these materials may be seen in Table 2. Scaffold materials generally fall into two categories: natural materials obtained from biological sources, or materials of purely synthetic origin. The synthetic hydrogels which have wide use in regenerative medicine include: PEGDA, GelMA, hybrid gels, etc. [87]. Some biological gels are derived from chitosan, fibrin, collagen, alginate, gelatin, and decellularized primary tissue. Each represents a unique design space at any scale [87].

**Figure 3:**
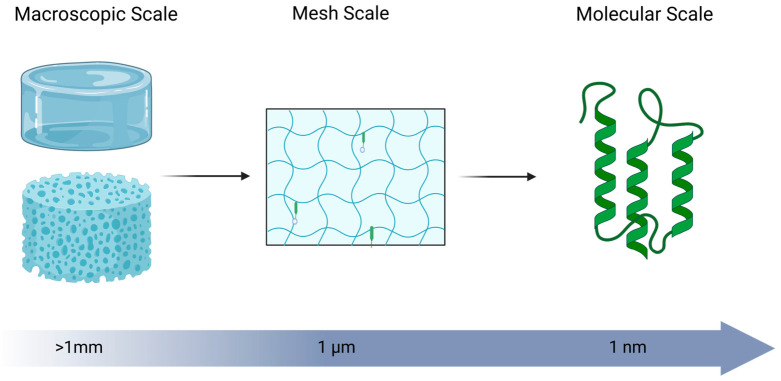
Schematic overview of the structural scales of hydrogels. Created in BioRender. Som, A. (2026) https://BioRender.com/3frmmqv.

Beyond the chemical backbones of the gels, the structural modifications, primarily chemical crosslinking, have the greatest effect on hydrogel properties. Higher degrees of crosslinking directly affect viscosity, retention, degradation, release, and hydrogel mesh size, (Figure 3), as well as mechanical stiffness, permeability, and the transport of bioactive molecules [11]. These properties directly influence cellular infiltration, nutrient diffusion, and release kinetics of encapsulated therapeutics. In the liver, both parenchymal and non-parenchymal cells contribute to regeneration necessitating both mechanical support and permeability to avoid impairing mass transport or creating local hypoxia [87,104]. Controlled degradation through hydrolytic or enzymatic pathways further enables temporal matching of material persistence with regenerative processes.

Biochemical composition plays another critical role in the regenerative capacities of these biomaterials. Materials presenting bioactive ligands or tissue-specific cues can modulate cell adhesion, phenotype, and function, enhancing integration with host tissue [104]. Considering this, liver tissue-derived dECM hydrogels represent a particularly promising class of biomaterial for liver regeneration. These materials are generated directly from primary human tissue treated to remove living cells and so retain organ-specific extracellular matrix (ECM) components and signaling molecules that were already supporting hepatocyte viability and function [104,105]. Liver dECM hydrogels have been widely used in three-dimensional culture systems, including bioprinting and microphysiological liver models, where they provide a better microenvironment than synthetic gels. In vivo studies further demonstrate their regenerative potential, with dECM-based scaffolds promoting hepatocyte proliferation, bile canaliculi formation, and reductions in liver fibrosis, alongside improvements in functional markers such as albumin and urea production in rodent models [104].

Some consideration must be taken that the microenvironment of a diseased liver may differ from that of a healthy liver. This can be used advantageously to design hydrogels that selectively release their contents in the target environment. Chronic inflammation in the liver results in a greater concentration of reactive oxygen species, which can stimulate the dissociation of bonds in a modified hydrogel such as boronic ester bonds [106]. Though liver disease can present with systemic acid-base abnormalities, they tend to balance each other in stable cirrhosis due to competing acidifying factors (bicarbonate loss, dilutional hyponatremia) and alkalinizing factors (respiratory alkalosis, hypoalbuminemia) [107]. Additionally, the diseased liver microenvironment is indicated to match physiological pH by CEST MRI showing the contrast between the acidic pH of hepatocellular carcinoma (HCC) tumors and the normal pH of the surrounding liver, which is cirrhotic in 80% of HCC cases [108,109]. Therefore, the use of hydrogels with pH-dependent functional groups or chemical crosslinks may be limited to differential delivery between cancerous and other diseased liver tissue [110,111].

Together, structural tunability, controlled degradation, biochemical signaling, and microenvironment compatibility form the basis for rational biomaterial selection in liver regeneration. In the context of intra-arterial delivery, vascular shunting (e.g., arterioportal or arteriovenous shunts) can reduce local retention and increase the risk of off-target distribution. Accordingly, particulate delivery systems often require sufficient size to ensure hepatic retention, as particles below ~40 µm may pass into the systemic circulation, while clinically used embolic materials are typically in the tens-of-microns range (e.g., ~40–120 µm or larger) to promote localization [112]. The following sections build upon this framework by examining specific material platforms and delivery strategies that leverage these principles for localized and controlled therapeutic intervention.

## 5. Released Regenerative Agents for Enhanced Liver Regrowth

**Figure 4:**
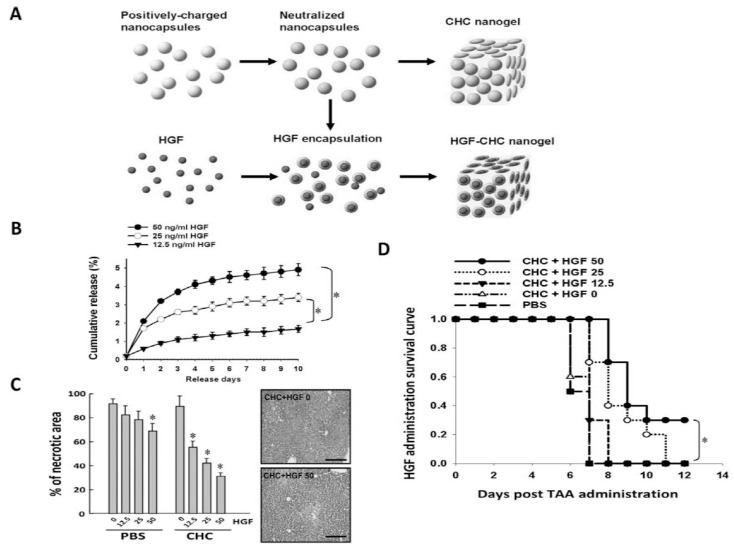
Characterization of HGF-CHC. (**A**) Design of the nanocapsule structures formed by the amphiphatic CHC hydrogel. (**B**) Left: dose-dependent HGF release. Effect of HGF-CHC delivery on hepatic necropsy and necrotic area in a TAA (200 mg/kg)-injured liver. Right: representative H&E images comparing the effect of CHC alone and CHC containing 50 ng/mL HGF. (**C**) Comparison of the efficacy of HGF using either CHC or PBS as delivery vehicles on the hepatic necrotic area in a TAA-injured murine liver. (**D**) Dose-dependent survival of recipients of HGF delivered by CHC. (**B**) * *p* < 0.05 versus 12.5 ng/mL HGF. (**C**) * *p* < 0.05 versus 0 ng/mL HGF with corresponding treatment (PBS or CHC). (**D**) * *p* < 0.05 versus 0 ng/mL HGF [116].

HGF and other cytokines critical to liver regeneration have very short half-lives, often lasting only minutes, which makes direct injection impracticable [113]. Hydrogels offer a solution by enabling the protective, localized, sustained, and stimulable release of bioactive molecules [114]. These tunable characteristics allow hydrogels to serve as reservoirs for bioactive molecules and cells [11]. In one study, rats receiving intraperitoneal injections of gelatin microspheres loaded with HGF showed reduced fibrosis and improved liver architecture after three weeks, compared to those treated with HGF or vehicle control alone [115]. Another approach is co-delivery of HGF with other regenerative treatments. Chiang et al. delivered HGF via a chitosan hydrogel with dental pulp-derived induced pluripotent stem cells (iPSCs) to mice with acute liver failure (ALF). Mice receiving both HGF and iPSCs had significantly improved survival compared to those receiving HGF or iPSCs alone following thioacetamide-induced injury (Figure 4) [116]. In healthy tissue, HGF diffusion is limited by ECM, and decellularized ECM hydrogels retain this property to enhance HGF potency in cell therapy co-delivery. For example, a porcine liver ECM derived hydrogel used to deliver hepatocytes and HGF achieved ~87% HGF immobilization within the gel and 18% transplanted cell survival, outperforming collagen gels in the same parameters (18% and 0.4%, respectively) [117].

An alternative strategy for longer-term biologically derived mitogen generation is through mRNA gene therapy. In one study, lipid nanoparticles (LNPs) were used to deliver nucleoside-modified mRNAs encoding HGF and EGF. A single intravenous injection resulted in protein expression lasting approximately three days, leading to improved ALT levels and enhanced liver regeneration in mice with acute and chronic liver failure [118]. Another study combined LNP-mediated delivery of HGF/EGF mRNAs with primary human hepatocyte (PHH) transplantation, resulting in improved PHH engraftment and enhanced liver function [24].

**Figure 5:**
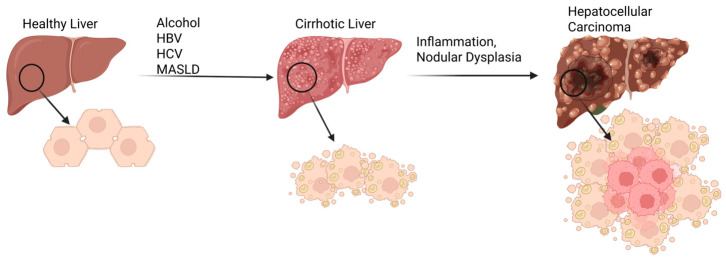
This is a basic representation of the formation of HCC within a failing liver. Beige cells represent hepatocytes with intracellular lipid accumulation, while pink cells represent malignant tumor cells. Darkened regions within the liver indicate areas of tissue damage and tumor formation. Inflammation from the damage of a healthy liver can cause nodular dysplasia of the hepatocytes that can progress into HCC. This is especially problematic because stimulating regeneration of tissue within a damaged liver would also stimulate regeneration of any HCC. Created in BioRender. Som, A. (2026) https://BioRender.com/ov2nz3c.

Significant challenges remain in the incorporation of released mitogen therapies. Precise control of release rates is essential to mimic physiological conditions, maximize therapeutic efficacy, and minimize cancer regrowth. Additionally, even in healthy liver, the overstimulation of a mitogen-like HGF can lead to uncontrolled tissue proliferation and HCC (Figure 5). This risk is especially present in patients with chronic liver disease who experience sustained cycles of injury and regeneration, with cirrhotic patients exhibiting an annual hepatocellular carcinoma incidence of approximately 1–8% depending on etiology [119,120]. This underscores the need for the tight regulation of growth factor dosing, release kinetics, and localization. Advancing hydrogel formulations and delivery strategies will be key to fully harnessing the regenerative potential of HGF and other growth factors in clinical settings.

## 6. Anatomic Considerations for Biomaterial Delivery

**Figure 6:**
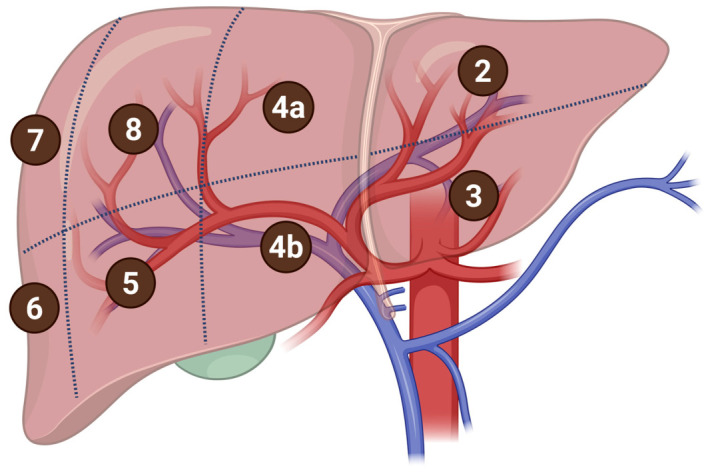
Segmental liver anatomy showing dual blood supply and independent functional segments numbered 1–8 (segment 1 not pictured). Red vessels represent branches of the hepatic artery while blue vessels represent branches of the portal vein. Created in BioRender. Som, A. (2026) https://BioRender.com/5fp3k5i.

Regenerative therapies can be precisely targeted in the liver through image-guided vascular delivery, taking advantage of its high vascularization from the dual blood supply. The dual blood supply includes oxygen-rich blood from the hepatic artery and nutrient-rich, oxygen-poor blood from the portal vein. In addition to the unique vascular architecture, the liver is traditionally organized into eight functional segments that can be selectively targeted, shown in Figure 6 [121]. Image-guided techniques allow for selective access to different vascular territories, allowing for precise interventions tailored to segmental anatomy. Such approaches support intralesional, perilesional, or endovascular delivery depending on the location and perfusion characteristics of the lesion.

Existing liver therapies currently employ techniques that require these routes of administration. Direct injection into liver parenchyma is common for tumor ablations and liver biopsies and can similarly be used to target a specific lesion for regeneration [122]. Arterial routes are often accessed for localized therapies that target a specific lesion, such as transarterial tumor embolization for cancer treatments [123]. Portal venous systems tend to be used for treatments further upstream with broader targets such as portal vein embolization to prepare for liver resection, although branches of the portal vein still supply individual segments of the liver [124]. While the portal vein can be directly accessed percutaneously through the abdomen or from a vessel that feeds it such as an ileocolic venous branch or the splenic vein [125,126], the arterial supply is accessed more peripherally with an arterial catheter entering at a radial artery or femoral artery [127]. However, the highly vascularized architecture of the liver presents a major challenge for hydrogel-based intrahepatic injection, as injected materials may enter the vasculature and result in off-target embolization or systemic washout. To mitigate this risk, both injection parameters (e.g., pressure and flow rate) and material properties (e.g., viscosity, shear-thinning behavior, and rapid gelation kinetics) must be carefully controlled to promote local retention [128].

A potential alternative route is via intraperitoneal injections, which are commonly used in murine studies for liver regeneration (Table 2). They are not commonly used for liver-specific treatments in humans but have been proposed in emerging clinical trials outside of the USA for regenerative therapies [129,130]. This route provides direct access to the peritoneal cavity, enabling regional delivery to abdominal organs, including the liver, while avoiding more invasive surgical implantation and reducing the complexity associated with intravascular or intrahepatic administration [131].

As these treatments are intended for those with liver failure, considerations must be made of a patient’s physiological conditions when determining paths to deliver treatment. Some of the hallmarks of liver disease, namely portal hypertension and coagulopathies, increase the risk of bleeding with the procedures listed above [132]. Splenic vein access can decrease the bleeding risk present with portal vein injections in the case of portal hypertension, and cell therapies implanted within the spleen have been shown to migrate to the liver [126,133]. Further progression of liver disease can also lead to the development of ascites, which complicates intraperitoneal and intra-parenchymal injection due to fluid buildup in the peritoneal cavity that can limit the uptake and implantation of regenerative therapies as well as an increased bleeding risk. Finally, HCC can cause damage, necessitating regenerative treatment, and due to the tumor preference for arterial blood, this may disqualify arterial routes of administration.

## 7. Engineering Constraints for Biomaterial Platforms

**Figure 7:**
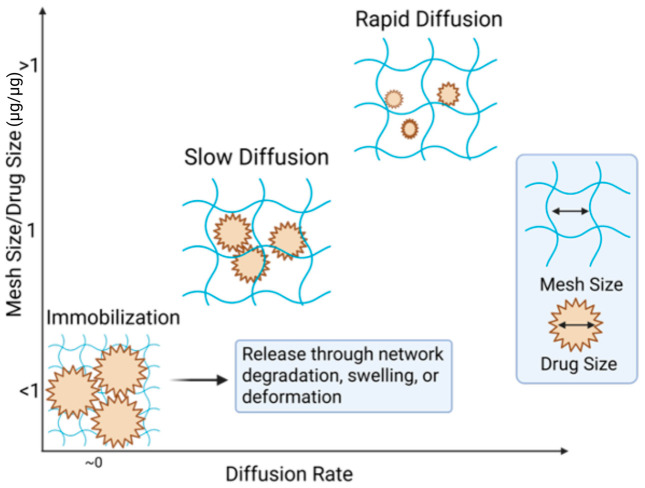
This is a schematic representation of hydrogel structure and its effects on release mechanisms and rates. Created in BioRender. Som, A. (2026) https://BioRender.com/eibtwuu.

Controlled release from hydrogel-based biomaterials arises from the interplay between network architecture, cargo size, and material degradation, as schematically illustrated in Figure 7. When the characteristic mesh size of the polymer network exceeds the size of the encapsulated biomolecule, rapid diffusive release can occur. As mesh size approaches the hydrodynamic diameter of the cargo, transport becomes increasingly hindered, resulting in slower, diffusion-limited release. When the mesh size is smaller than the cargo, biomolecules may be effectively immobilized within the network until changes in the material structure enable release [11].

As shown in Figure 7, initially immobilized networks can be triggered to release through swelling, deformation, or degradation-driven mesh expansion [11]. In swelling- or diffusion-controlled systems, release is governed by the relationship between network mesh size and therapeutic size. For example, Parlow et al. demonstrated that diffusion coefficients of macromolecules (4–150 kDa dextrans) decrease systematically as molecular size approaches the hydrogel mesh size, reflecting steric hindrance within the network [134]. In hydrogels formed from degradable polymers, bond cleavage within the network increases mesh size over time, enabling delayed or sustained release [11]. As a representative case, Liu et al. showed that, when the mesh size is comparable to the size of the encapsulated therapeutic, diffusion is restricted, and release is delayed until degradation-driven mesh expansion permits transport [135]. In contrast, synthetic polyesters such as PLGA primarily exhibit degradation-controlled release, where hydrolytic cleavage of ester bonds leads to molecular weight reduction and bulk erosion [136,137,138]. For example, as described by Makadia and Siegel, drug release from PLGA systems occurs through a combination of diffusion and bulk erosion, producing a characteristic biphasic profile with an initial burst followed by sustained release. Reported systems, such as ciprofloxacin-loaded PLGA 50:50 implants and risperidone formulations, demonstrate controlled release over timescales ranging from weeks to several months, depending on polymer composition and molecular weight [98]. Across both natural and synthetic biomaterials, biodegradation kinetics—whether enzymatic or hydrolytic—are therefore central determinants of release behavior and in vivo residence time [138].

For injectable hydrogels, the careful design of material properties is essential for injectability and site adherence. These systems must retain low viscosity during needle extrusion, while rapidly gelling and achieving functional geometry once delivered to the target site [139,140].

Extravascular hydrogels can be thermo-responsive or shear thinning, allowing them to liquify during injection and solidify into a gel in the body. This transition is essential for their localization and function at extravascular sites such as tumors or the organ’s surface [141,142]. Thermo-responsive systems undergo gelation in response to physiological temperature, enabling stable in situ depot formation under relatively constant body conditions. Chatterjee et al., for example, demonstrated the utility of Pluronic F-127-based thermosensitive systems for sustained transdermal release [143]. In contrast, shear-thinning hydrogels rely on reversible network interactions that enable flow under applied stress and recovery upon stress removal, making their behavior inherently dependent on local mechanical and flow conditions [144]. This sensitivity can also necessitate additional stabilization strategies, as shear-thinning systems may exhibit reduced structural integrity without secondary crosslinking [145]. When designed with shear-thinning properties, these hydrogels can be hand-injected through smaller needle gauges, improving patient comfort during administration [140,146]. Avery et al. developed such shear-thinning systems and demonstrated their compatibility with clinical catheters and needles across various delivery settings [147].

Intravascularly, hydrogels must withstand significant hydrostatic pressures to reach and adhere to their target site [148]. Lee SY et al. developed a catheter-injectable embolic hydrogel for HCC with controlled gelation time, tunable rheology, and the ability to deliver multiple therapies [149]. This system effectively navigated distal arterioles, conformed to the vessel architecture, and delivered doxorubicin (DOX), glucose oxidase, and MnO_2_ directly to the tumor, resulting in a 54% increase in tumor necrosis compared to no treatment. Similarly, Avery et al. engineered an injectable shear-thinning biomaterial for endovascular embolization, demonstrating its ability to conform to complex vascular spaces and remain locally retained [147]. Collectively, these strategies provide a foundational framework for hydrogel adaptation in regenerative medicine, enabling sustained therapeutic release while minimizing off-target effects.

**Figure 8:**
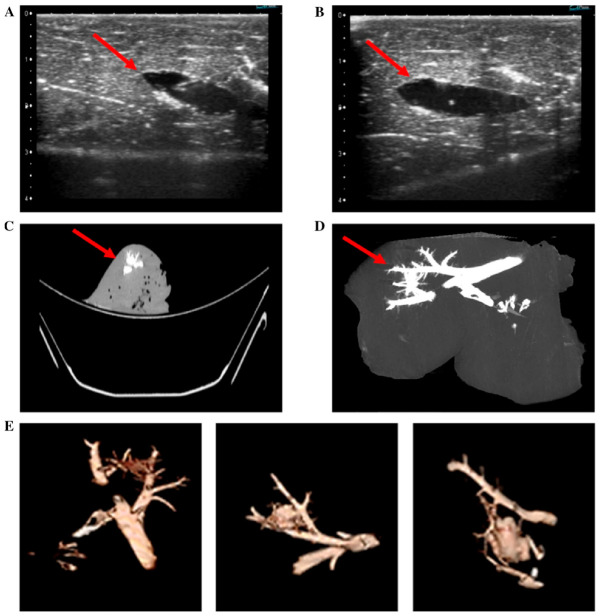
Ex vivo tissue administration of the radiopaque thermo-responsive hydrogel can be visualized using CT and US. Representative (**A**,**B**) US and (**C**,**D**) CT images of radiopaque hydrogel distribution in ex vivo tissue and (**E**) CT images of radiopaque hydrogel pooling at parenchymal injection site (arrow), following which, it spreads through vascular and biliary channels and molds to the shape of the liver vasculature via a phase transition [140].

Radiopacity and echogenicity are commonly used methods for tracking hydrogel localization and distribution (Figure 8) [140,141,142,149]. These features can be engineered through the incorporation of radiographic and echogenic contrast agents, providing imageability for the verification of the injection site, in vivo behavior, and degradation, which is critical for dose quantification and minimizing toxicity [141,142]. However, the incorporation of iodinated contrast agents presents a significant design challenge, as imaging strategies must be carefully engineered to avoid altering hydrogel physicochemical and transport properties [150]. The relatively high concentrations required for radiographic visibility (often 1–100 mg/mL) can disrupt hydrogel network structure, mechanical behavior, and diffusion characteristics, potentially impacting drug release kinetics and overall material performance [151]. In addition, contrast agents that are not chemically integrated may diffuse from the hydrogel over time, necessitating covalent incorporation strategies to maintain localization and imaging fidelity [152]. As such, contrast integration must be considered early in the material design process, as addition later in development can be impractical due to the need for re-optimization and additional safety validation.

The addition of imaging agents is important for the verification of the proper degradability and physiochemical properties of the hydrogels in vivo [87]. For example, Delgado et al. developed a poloxamer-based hydrogel that was visible under both ultrasound and X-ray by incorporating iodine and microbubbles via gentle stirring [142]. The microbubbles were prepared in a saline/glycerol/polyethylene glycol mixture and added to the gel via pipette, where they enhance imaging contrast and enable clearer differentiation of the material from surrounding tissue. MRI is also a viable imaging modality; however, it typically requires contrast agents such as fluorine, gadolinium, or ferromagnetic particles which are not naturally present in the human body. Kolouchova et al. demonstrated that hydrogels with fluorine-containing polymers can be effectively visualized using MRI and exhibit controlled biodegradation while remaining well-tolerated in vivo, with evidence of tissue integration and support of surrounding tissue growth rather than pathological response [153]. This ability to visualize hydrogels in real time is vital for the effective design and clinical translation of injectable biomaterials.

Different imaging modalities offer distinct advantages and limitations. X-ray-based methods such as CT provide high spatial and temporal resolution, enabling real-time anatomical imaging at relatively low cost, which has contributed to their widespread adoption [150]. A drawback, however, is the reliance on heavy-element contrast agents, the most clinically relevant being iodine, which can pose toxicity risks to patients [154]. Ultrasound, in contrast, is inexpensive, portable, and radiation-free, making it widely accessible for real-time imaging [155]. Yet, ultrasound generally offers lower resolution than CT and struggles to distinguish tissues with similar densities [156]. Furthermore, the microbubble contrast agents commonly used in ultrasound are prone to diffusion or rupture over time, diminishing image quality [157].

Translating injectable hydrogels into clinical practice is quite feasible, as the image-guided techniques required for their delivery are similar to many procedures already in use [158]. Implementing these systems would likely involve minimal additional training or cost. The liver, for example, can be accessed through the hepatic artery or portal vein, routes commonly used in procedures like embolization or portal vein thrombolysis. These are minimally invasive and generally considered low-risk, making them ideal pathways for targeted hydrogel delivery.

## 8. Liver Tissue Engineering Using Hydrogels

Liver tissue engineering marks a major step forward in regenerative medicine, with hydrogels providing an ideal platform by closely mimicking the liver’s microenvironment [87,104,159]. These scaffolds can encapsulate hepatocytes, endothelial cells, and ECM components, supporting cell attachment, growth, and function while offering a protective immunological barrier to fragile cells. In one study, human hepatocyte organoids (hHOs) encapsulated in peptide-modified PEG hydrogels maintained function for over three months in mice and rescued four out of six pigs with no immune rejection despite xenotransplantation [160]. Human hepatocyte microbeads (HMBs) preserved albumin and urea production for seven days, even in human ascitic fluid. Similarly, rat hepatocyte microbeads (RMBs) led to 100% survival in rats with ALF compared to 50–80% in controls. The calcium-alginate coating provided immune protection and maintained cell viability without immunosuppression [161].

Despite their compatibility with the liver, a few challenges limit the widespread application of hydrogels. These include cell damage from shear stress during injection, cytotoxicity associated with certain crosslinking methods, and limited macroporosity, which can hinder long-term tissue integration [162].

**Figure 9:**
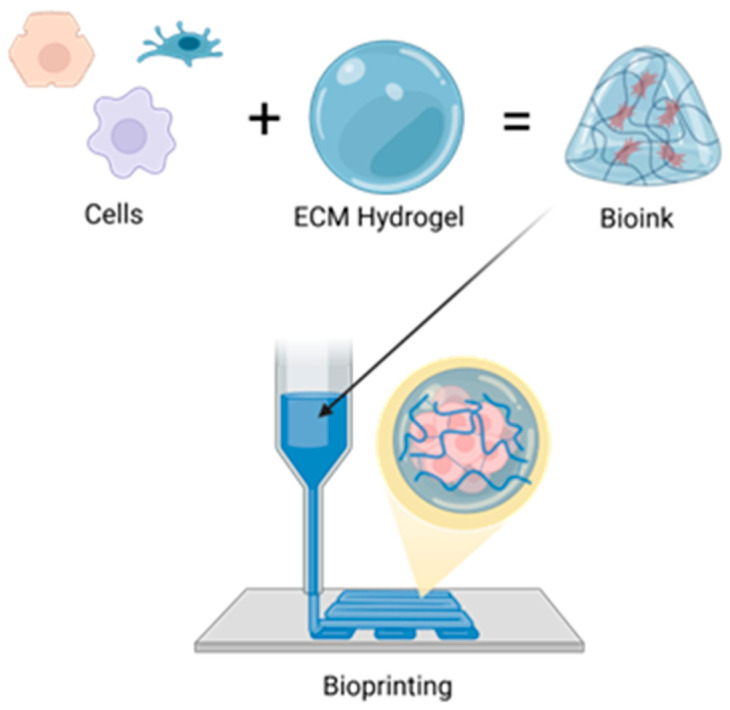
Representative bioprinting schematic. A bioink made of an ECM mimicking hydrogel is laden with cells and then extruded as shown here, or alternatively ink jet-printed, or cured in place using stereolithography to manufacture functional microphysiological systems. Created in BioRender. Som, A. (2026) https://BioRender.com/zkz7vzo.

Beyond serving as 3D scaffolds that mimic the liver ECM and support hepatocyte viability and organization, hydrogels are now integral to microfluidic systems and bioprinting technologies used to build complex liver models for drug testing [94]. ECM-based scaffolds such as Matrigel and collagen hydrogels are commonly used in organoid-on-chip systems. Techniques like 3D printing, including approaches such as FDM printing and digital light processing (DLP), make it possible to not only encapsulate cells in a matrix, but allow the precise placement of cell-laden bioinks to mimic natural structure [163]. A schematic representation of one such approach, extrusion bioprinting, is depicted in Figure 9, where an ECM-based hydrogel is loaded with cells to create the bioink that can then be arranged to form functional microphysiological systems. In this example, the bioink must simultaneously support cell encapsulation, extrusion through a printing nozzle, and rapid structural stabilization after deposition [164]. These requirements can be directly controlled through crosslinking strategies: shear-thinning, physically crosslinked systems facilitate extrusion and cell protection during printing, and secondary or covalent crosslinking steps often enhance post-printing mechanical stability and fidelity [164]. The degree of crosslinking is also vital for these systems. Loosely crosslinked bioinks would flow easier through an extruder but would be limited in their capacity to form large structures without collapse [164]. In this way, the crosslinking architecture can determine the cellular microenvironment of the microphysiological systems as well as the final geometry of the fabricated systems.

**Figure 10:**
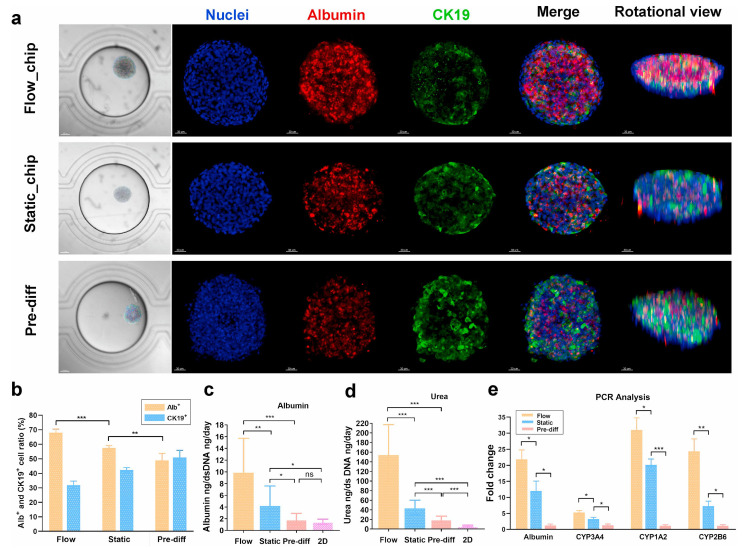
Functional characterization for in situ differentiated HepaRG organoids (flow and static condition), and pre-differentiated HepaRG spheroids. Blue staining indicates nuclei, red staining indicates albumin expression, and green staining indicates CK19 expression. Merged images show colocalization of the markers. (**a**) Staining with albumin and CK19 to visualize the hepatocyte and cholangiocyte/progenitor cell expression and distribution in HepaRG organoids/spheroids generated from three different culture conditions. Scale bar is 30 μm. (**b**) Quantification of the ratio of albumin+ and CK19+ cells in the organoids/spheroids. (**c**) Albumin secretion and (**d**) urea synthesis of the HepaRG organoids/spheroids generated from three different culture conditions, and 2D cultured HepaRG, were evaluated using ELISA assays. (**e**) Quantification of albumin and CYP gene expressions using qPCR, with data normalized to that in the pre-differentiated spheroids. All the experiments were performed in triplicate and repeated at least three times. *, **, and *** denote statistically significant differences with *p* values less than 0.01, 0.005, and 0.001 respectively. This figure from Teng et al. is a representation of how microfluidic devices can be used to mimic the natural processes in human organs, highlighting their potential use in testing new treatments [165].

Liver-on-a-chip (LoC) testing is the most advanced form of these methods used to model specific liver diseases, that could benefit from these advances. For instance, Teng et al. developed the SteatoChip, a 100-well microfluidic device modeling NAFLD (now MASLD), using hydrogel-encapsulated HepaRG cells to form 3D cellular clusters in each well, offering a scalable alternative to animal models [165]. Freag et al. created a NASH-on-chip model using a collagen matrix with co-cultured hepatocytes, Kupffer cells, hepatic stellate cells, and liver sinusoidal endothelial cells under dynamic perfusion, achieving stable liver function for up to two weeks. They also found that induced lipid accumulation was reduced with the application of elafibranor along with inflammatory markers and profibrotic markers [166]. While these organ-on-chip systems can replicate liver function, they still face challenges in modeling immune and microbiome interactions [167]. LoC platforms range from static to perfused 3D-printed designs, and standardization across models could enhance both research and commercial development [168]. Functional characterization of engineered liver constructs under static and perfused conditions is shown in Figure 10, illustrating albumin secretion, urea synthesis, and gene expression profiles that validate hepatocyte differentiation and maturity within microfluidic systems [165].

Recent advances in 3D bioprinting have enabled precise placement of cells and biomaterials, helping recreate liver tissue architecture with greater accuracy [169]. 3D bioprinting may enable the construction of liver tissue models for clinical research, drug testing, and regenerative therapies. Various printing techniques, including inkjet, extrusion, and stereolithography, are used to create liver tissue models. These models have demonstrated key liver functions such as urea synthesis, albumin secretion, and drug metabolism [170]. Microfluidic devices further support tissue development by controlling nutrient delivery, waste removal, and shear stress, promoting functional maturation of engineered liver constructs [171]. By combining precise bioprinting with microfluidic technologies, researchers have been able to get closer to replicating some of the structure and function of native liver lobules [172]. Beyond serving as research platforms, the knowledge gained from organ-on-a-chip systems can directly inform the design of in vivo injection strategies and hydrogel-based delivery platforms making their consideration important in the pre-design phase of therapeutic development. In a recent study, bioprinting artificial blood vessels using endothelial cells within bioprinted liver tissue enabled the transplanted tissue to be directly connected to the inferior vena cava and portal vein in a rat model resulting in the rapid establishment of circulation through the transplanted tissue [173]. Despite these innovations, challenges remain such as achieving full tissue maturation, establishing vascular networks, and ensuring proper integration with host tissue. Long-term viability and transplant success are also major hurdles that must be addressed before clinical use becomes widespread.

## 9. Cell Therapies for Liver Regeneration Are Enhanced When Combined with Hydrogel Platforms

Stem cells hold great promise for liver regeneration due to their ability to differentiate into hepatocyte-like cells capable of albumin secretion, urea synthesis, and Cytochrome P450 expression [174]. Among the types explored, iPSCs and MSCs are most prominent. iPSCs, reprogrammed from somatic cells like dental pulp, are patient-specific, reducing the risk of immune rejection [175]. MSCs may aid liver repair by differentiating into hepatocytes and releasing regenerative factors and are valued for their anti-inflammatory and immunomodulatory effects [176,177]. Umbilical cord-derived MSCs, which express low MHC-I and lack MHC-II, may further minimize immune response [178]. In adults, MSCs can also be sourced from bone marrow, adipose tissue, and dental pulp, offering potential for autologous transplantation.

**Figure 11:**
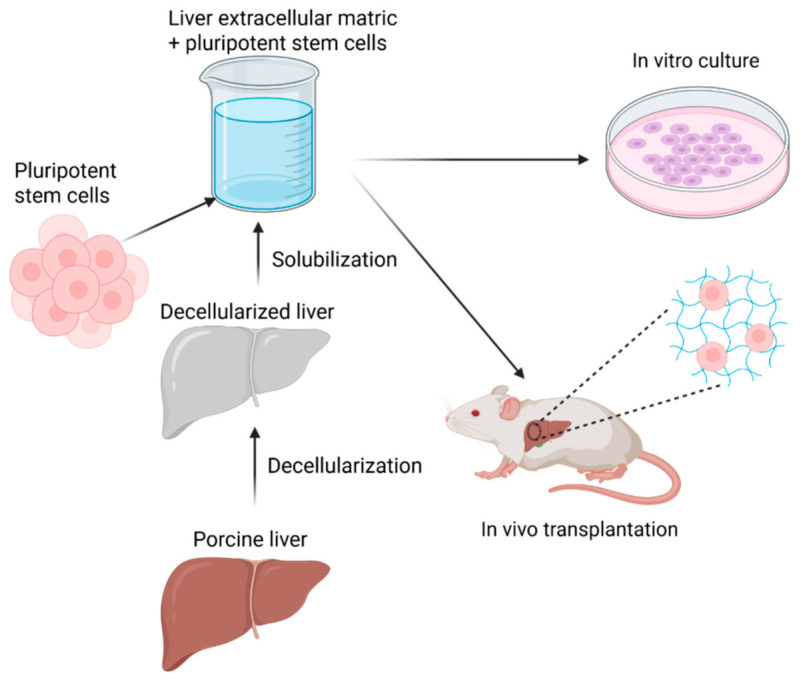
This is a schematic representation of using decellularized liver tissue for culturing pluripotent stem cells for in vitro cell culture experiments or in vivo experiments. Decellularized ECM is a hydrogel that can gel under physiological conditions, making it an ideal candidate for cell culture and in vivo experiments thanks to its innate biocompatibility. Created in BioRender. Som, A. (2026) https://BioRender.com/vezmjqz.

From a materials perspective, the performance of hydrogel-assisted cell therapies is governed by the chemical composition and crosslinked network architecture of the scaffold [11]. Natural polymers such as chitosan, collagen, and alginate provide bioactive motifs that facilitate cell adhesion and signaling, whereas synthetic polymers such as poly(lactic-co-glycolic acid) (PLGA) offer tunable degradation kinetics and mechanical strength [12]. Chitosan, a partially deacetylated polysaccharide composed of β-(1→4)-linked D-glucosamine units contains primary amine groups that enable ionic crosslinking, pH-responsive swelling, and chemical functionalization [103]. Collagen hydrogels form fibrillar networks through self-assembly of triple-helical domains and inherently present integrin-binding sequences that support hepatocyte attachment [179]. Alginate, a linear copolymer of β-D-mannuronic acid and α-L-guluronic acid residues, undergoes ionic crosslinking in the presence of divalent cations such as Ca^2+^, forming networks with tunable porosity [180]. In contrast, PLGA degrades through hydrolytic cleavage of ester bonds, allowing predictable bulk erosion and sustained release of encapsulated factors [98]. These chemical and structural differences directly influence stem cell retention, nutrient transport, matrix remodeling, and the kinetics of growth factor presentation within regenerating liver tissue [181]. Among these platforms, liver-derived decellularized extracellular matrix (dECM) hydrogels represent a biomimetic strategy, as they retain native liver-specific ECM proteins, glycosaminoglycans, and bound signaling cues that support hepatocyte function and lineage stability [104]. A schematic representation of this can be seen in Figure 11 below.

The differentiation of stem cells such as iPSCs and MSCs into hepatocytes requires tightly regulated signaling pathways, ECM interactions, and stepwise induction with specific growth factors to guide hepatic lineage commitment and maturation [174,182]. While undifferentiated stem cells can become hepatocytes under appropriate conditions, there is also a risk of differentiation into unintended cell types [177].

Stem cell therapies, without hydrogel-based delivery, have been tested in clinical trials for both ALF and ACLF. These include G-CSF-based strategies to mobilize bone marrow stem cells and direct stem cell transplantation [183]. G-CSF has shown potential in improving liver function, MELD scores, and survival in ACLF patients, with generally good safety profiles [184]. A double-blind trial reported increased CD34+ cell mobilization and higher 60-day survival with subcutaneous G-CSF, also noting its role in stem cell trafficking and immune activation [185]. Although G-CSF has shown some benefit in improving liver function and survival in ACLF, a multi-center study found limited efficacy and noted adverse effects, possibly due to its dual roles in regeneration and inflammation [186]. As opposed to G-CSF, meta-analyses of other trials using direct stem cell administration more consistently suggest that direct transplantation improves liver function and reduces liver injury [187,188].

**Figure 12:**
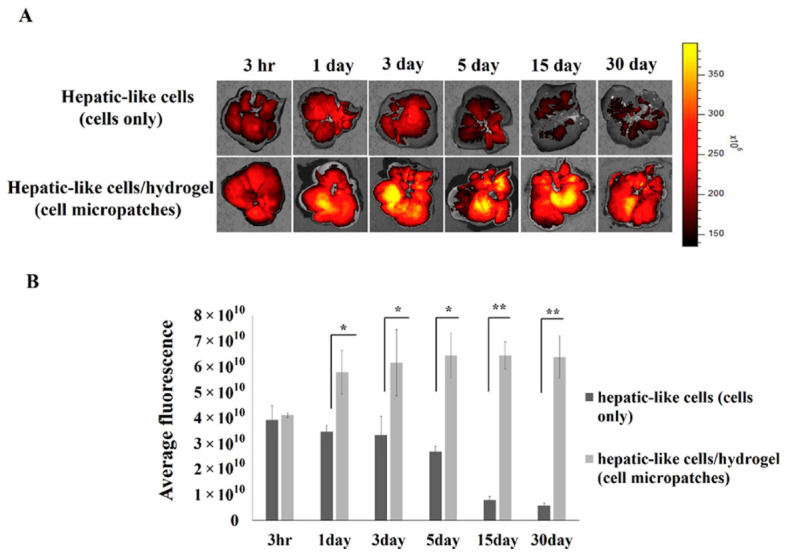
Liver localization of hepatic-like cells observed by IVIS. (**A**) Representative fluorescence images. (**B**) Quantification of fluorescence intensity of the rats liver after 3 h, 1, 3, 5, 15, and 30 days upon intravenous injection of DiD-labeled hepatic-like cells. Data as means ± STD with n = 3. * *p* < 0.05, ** *p* < 0.01 [191].

Hydrogel scaffolds enhance stem cell-based liver regeneration by improving cell retention at injury sites (Figure 12), supporting cell survival and proliferation, and guiding controlled differentiation [189]. By mimicking aspects of the native ECM, hydrogels facilitate essential cell-to-cell interactions. They also enable the co-delivery of stem cells with growth factors like HGF, further promoting cell viability [117]. Animal studies repeatedly show that hydrogel-mediated stem cell delivery leads to better outcomes compared to delivery without hydrogels [117,190,191].

Beyond native polymer selection, chemical modification strategies further expand the functionality of these biomaterials. Chitosan can be derivatized through quaternization, thiolation, or grafting of PEG to enhance solubility, reduce immunogenicity, and control degradation rates [192]. Collagen matrices may be crosslinked chemically with genipin or carbodiimide to increase mechanical stability and prolong in vivo residence time, though excessive crosslinking can impair cellular infiltration [193]. Alginate can be oxidized to introduce hydrolytically labile linkages, enabling partial degradability in mammalian tissues where native alginate is otherwise non-degradable [194]. PLGA properties are adjusted through lactic:glycolic ratio and molecular weight selection, allowing the precise tuning of degradation from weeks to months [100]. These modification strategies alter mesh size, stiffness, and degradation kinetics, which in turn regulate stem cell survival, differentiation cues, and the temporal presentation of regenerative signals [195]. Thus, rational chemical design of the biomaterial scaffold is central to optimizing cell-based liver regeneration therapies.

Another approach to biomaterial-mediated stem cell engraftment are carriers that directly transport cells to specific tissues. In a recent study by Mohan et al., mesenchymal stem cell (MSC) therapy was combined with polymer nanocarriers (NCs) complexed with specific adhesion molecules that targeted treatment to porcine livers that underwent radiofrequency ablation. Pigs treated with NC+MSC showed significantly greater liver regeneration, ~80% reduction in ablation cavity volume compared to those receiving MSCs without NCs getting only ~60% reduction in ablation cavity volume. However, no significant differences were observed in liver function, renal function, or complete blood counts between the groups at any point in the study [196].

Stem cell-derived exosomes have also shown protective effects in liver failure. These vesicles carry bioactive molecules that promote hepatocyte proliferation and suppress inflammation and apoptosis [197]. Exosomes can also be loaded with hard-to-deliver therapeutics, such as non-coding RNAs [198], and when embedded in hydrogels, benefit from sustained, localized delivery that enhances their regenerative potential [199].

While promising, stem cell delivery via hydrogels still faces key challenges. Major hurdles of poor cell viability and integration may be caused by the large size of stem cells relative to the small pore size of many hydrogel networks [189]. Vasculogenesis is also difficult to achieve and potentially requires the incorporation of a mixture of cell types [200]. Immune responses, even with autologous cells, along with ethical concerns and limited stem cell availability, also complicate the use of these methods [177]. In contrast, stem cell exosomes are smaller, less immunogenic, and easier to deliver, though they rely upon stimulating regeneration via endogenous cells in contrast to the self-renewal capacity of stem cells. Continued research is needed to overcome these limitations and optimize both cell- and EV-based strategies for liver regeneration.

## 10. Metallic Nanoparticles in Liver Regeneration

Metallic nanoparticles (NPs) have been growing in popularity for decades and show particular promise in treatments for liver regeneration due to their propensity to collect in the liver sinusoids [201]. This selective uptake of NPs within the liver can be leveraged to enhance liver regeneration following toxic or surgical injury. NPs have been shown to reduce oxidative stress, suppress inflammation, and stimulate hepatocyte proliferation, acting as either catalytic antioxidants (nanozymes) or as functionalized carriers for bioactive molecules or stem cells [14,202]. Across the studies discussed in this section, we discuss a range of metallic NPs including cerium oxide, manganese oxide, gold, and iron oxide NPs with reported sizes ranging from tens of nanometers to a few hundred nanometers. These NP systems had surface chemistries tailored to promote hepatic accumulation, catalytic activity, or improve cellular targeting.

Cerium oxide NPs can act as scavengers of reactive oxygen species (ROSs), helping to reduce oxidative stress in damaged livers [203]. In work by Cordoba-Jover et al., they found that their CeO_2_ NPs accumulated in the liver reaching concentrations of ~160 µg Ce/g liver tissue within 90 min and persisting there for up to 8 weeks following injection. In partial hepatectomy (PH) rat models, their particles showed marked improvements over controls, raising their Ki-67+ hepatocyte counts fourfold over the control the first day after PH, and they found rat models had an ~11% increase in their hepatic regenerative index when treated with CeO_2_ NPs [204]. Additionally, Carvejal et al. found that, in human HepG2 cell cultures, CeO_2_ NPs demonstrated a strong capacity to mitigate oxidative stress caused by hydrogen peroxide. Following treatment with 1.5 mM H_2_O_2_, cells treated with CeO_2_ NPs returned glutathione levels from 37% of basal levels up to 64%, and at 10 µg/mL concentrations, CeO_2_ NPs did not significantly affect baseline cell viability [205].

Manganese oxide NPs have shown very similar nanozyme activity to CeO_2_ NPs. In a paper by Wu et al., their MnO_2_ nanoflowers (NFs) efficiently decomposed 29% of H_2_O_2_ at 100 µg/mL and scavenged over 90% of free radicals, O_2_^−^, and OH^−^. Their NFs also accumulated in the liver of their mice models and had no increase in their ALT/AST levels in their control mice. Following acetaminophen-induced ALF mice models pretreated with NFs showed >60% lower ALT/AST when compared to control mice [206]. Similarly, Liu et al. showed that their Mn_3_O_4_ nanozymes decreased the ALT/AST levels in mouse models with acetaminophen-induced liver damage by >60%, nearing the levels of mice treated with NAC, the standard antidote for acetaminophen overdose [207].

Gold NPs (AuNPs) have also been shown to have antioxidant and anti-inflammatory properties as well as good biocompatibility, ease of bioconjugation, fibrosis prevention, and immune modulation through NF-κB and Nrf2 signaling [208]. The hepatoprotective nature of AuNPs was also highlighted in a paper by Garcia et al., who showed that AuNPs, when co-delivered with carvedilol (a beta blocker), normalized levels of albumin and AST in rats with methamphetamine-induced fibrosis [209].

Iron oxide NPs (IONPs) have several advantages over other metallic NPs. They are more biocompatible because they can be naturally metabolized within the body, and their magnetic properties make them especially desirable for cell-labeling and targeted delivery methods. Their tracking/targeting capabilities and capacity for functionalization as active modifiers for regeneration make them an ideal dual-use platform for liver regeneration methods [210]. Lai et al. took advantage of these properties in their work by coupling superparamagnetic iron oxide (SPIO) with MSCs in rats with CCl_4_ induced liver fibrosis. Using their SPIO NPs, they transfected ad-HGF into the MSCs for group A whereas group B received only MSCs labeled with SPIO and GFP. Mice in group A showed 2.2× more Ki-67 hepatocytes 15 days after treatment and ~35% lower ALT levels. In vitro, their SPIO-HGF-MSCs also showed 2× higher oxidative resistance compared to SPIO-GFP-MSCs when exposed to H_2_O_2_ [211]. Additionally, it has been found that SPIO labeling is safe for MSCs and allows for the noninvasive MRI tracking of cell therapies [212,213]. Shen et al. were able to show that hepatocytes loaded with magneto-plasmonic Fe/Au nanoclusters could be guided with magnetic fields for hepatic targeting to improve cell engraftment in mice models receiving hepatocyte transplantations. Their mouse models showed 3–5× faster liver regeneration post-transplantation in mice receiving the modified hepatocytes, and liver repopulation efficiency was more than 10× compared to non-modified hepatocytes [214].

Metallic nanoparticles offer multiple different properties that can be harnessed to mitigate liver damage and improve hepatic regeneration. Cerium oxide and manganese oxide NPs act as catalytic antioxidants, scavenging reactive oxygen species and accelerating hepatocyte regeneration after acute injury. AuNPs also provide antioxidant properties as well as antifibrotic properties through the activation of Nrf2 and suppression of TGF-β/NF-κB pathways. In contrast, IONPs are primarily used as biocompatible and metabolizable platforms for stem cell delivery and MRI tracking. Their magnetic properties also open the door for guided cell transplantation. With the benefits of these therapies in mind, it is important to note that the preferential buildup of these particles within the liver raises concerns over potential cytotoxicity and clearance of the particles, particularly for the Au, Ce, and Mn that cannot be metabolized.

## 11. Preclinical Studies of Biomaterial-Mediated Growth Factor and Cell Therapy Delivery in Liver Regeneration

Preclinical studies have demonstrated that the biomaterial-mediated delivery of growth factors and/or cell therapies can effectively enhance liver regeneration by providing localized and sustained presentation of mitogenic and pro-regenerative signals. Injectable hydrogels and nanoparticle-based carriers have been used to deliver factors such as HGF, EGF, gene therapies, and hepatocytes or progenitor cells directly to injured liver tissue, resulting in improved hepatocyte proliferation, reduced fibrosis, and enhanced functional recovery compared to bolus administration. These platforms address key limitations of soluble growth factor therapy, including rapid degradation, poor tissue retention, and systemic toxicity, as well as limitations in cell engraftment in the absence of hydrogels. Collectively, findings from rodent models of acute and chronic liver injury underscore the therapeutic potential of these interventions as a minimally invasive and clinically adaptable strategy for hepatic regeneration. Representative injectable hydrogel and nanoparticle-based regenerative strategies evaluated in preclinical models are summarized in Table 3.

## 12. Conclusions and Future Work

Advances in liver regeneration have evolved from simple biomimetic concepts toward integrated platforms that combine biomaterials, biochemical signaling, and cellular therapies. Among these approaches, injectable hydrogel systems have emerged as versatile tools for addressing the biological and clinical challenges of hepatic repair. By enabling localized and sustained delivery of regenerative cues such as HGF, EGF, nucleic acids, stem cells, extracellular vesicles, and organoids, hydrogels amplify endogenous regenerative pathways while minimizing systemic exposure. Complementary developments in tissue-engineered scaffolds further demonstrate that hydrogel-based matrices can support transplanted cells long enough to achieve engraftment and functional contribution, offering a bridge between acute injury and durable recovery.

In parallel, metallic nanoparticle platforms have expanded the regenerative toolbox by introducing catalytic, antioxidant, and imaging capabilities that address oxidative stress, inflammation, and fibrosis. Cerium oxide and manganese oxide nanozymes mitigate reactive oxygen species in acute injury settings, while gold nanoparticles modulate inflammatory and fibrotic signaling. Iron oxide nanoparticles provide additional advantages as biodegradable, clinically familiar materials that enable stem cell tracking, targeting, and image-guided monitoring.

A defining strength of biomaterial-enabled liver regeneration lies in its alignment with interventional radiology. Image-guided delivery using ultrasound, computed tomography, and fluoroscopy enables the real-time visualization of material placement, distribution, and retention, improving procedural accuracy and dose control. Catheter based access via the hepatic artery, portal vein, or splenic circulation leverages delivery routes already established for embolization, ablation, and regional chemotherapy, allowing regenerative therapies to integrate into existing clinical workflows.

Despite strong preclinical evidence, most biomaterial-enabled regenerative strategies remain at an early stage of translation. Key challenges include achieving predictable in vivo behavior under physiologic blood flow, ensuring vascular integration within engineered tissues, and preventing spatially uncontrolled regenerative signaling that could increase oncogenic risk. Long-term biodegradation, clearance, and immunologic safety must also be rigorously evaluated, particularly for inorganic nanoparticles that preferentially accumulate in the liver. Standardization of dosing, delivery routes, and imaging metrics will be essential to enable meaningful cross-study comparisons and accelerate regulatory progress. Additionally, increased treatment complexity—arising from multi-component formulations, specialized delivery techniques, and imaging requirements—can present a significant barrier to clinical adoption, both from a practical implementation standpoint and due to associated economic costs.

Future efforts should prioritize imageable, catheter-deliverable hybrid platforms that integrate injectable hydrogels with nanoparticle-based targeting, catalytic, and contrast functions. Such systems would enable spatiotemporally controlled regeneration with real-time image-guided placement, intraprocedural verification, and longitudinal monitoring of therapeutic persistence and response. Emerging clinical initiatives highlight the feasibility of this approach and underscore the importance of continued interdisciplinary collaboration, though they are yet to begin patient enrollment [130].

In summary, biomaterial-enabled and image-guided regenerative therapies represent a clinically realistic pathway for restoring liver function without whole-organ replacement. By leveraging the precision, scalability, and established infrastructure of interventional radiology, these approaches have the potential to advance liver regeneration from experimental promise to a minimally invasive therapeutic strategy with meaningful impact for patients who currently lack viable treatment options.

## Figures and Tables

**Table 2:** An overview of some of the different scaffold materials common to liver regeneration using hydrogels and other polymeric systems.

Material	Abbreviation	Natural/Synthetic	Key Functional Groups	Biocompatibility	Degradation Products	Approximate Degradation Rate Under Physiological Conditions	Citations
Decellularized liver ECM	dECM	Natural	Mixed ECM proteins (–NH_2_, –COOH), GAGs (–SO_3_^−^, –COO^−^)	Excellent (tissue-specific cues)	Peptides, amino acids, GAG fragments	Days–weeks	[88,89]
Collagen (Type I)	-	Natural	Peptide backbone (–NH_2_, –COOH)	Excellent (adhesive bioactive)	Amino acids, peptides	Days–weeks	[87,90,91]
Gelatin methacrylate	GelMA	Natural (modified)	–NH_2_, –COOH; methacrylate (–C=C–)	Excellent (cell adhesive, bioactive)	Peptides, amino acids	Days–weeks	[92,93]
Hyaluronic Acid	HA	Natural	–COO^−^, –OH	Excellent (native ECM component)	Oligosaccharides	Days–weeks	[87,94]
Poly(ethylene glycol diacrylate)	PEGDA	Synthetic	Acrylate (–C=C–), –OH	Excellent (bioinert, tunable)	PEG fragments	Tunable (days–months, linker-dependent)	[94,95]
dECM-GelMA hybrid hydrogels	-	Hybrid	Mixed ECM + methacrylate	Excellent (bioactive + tunable)	Mixed (ECM + peptides)	Tunable (days–weeks)	[96,97]
Poly(lactic-co-glycolic acid)	PLGA	Synthetic	Ester linkages (–COO–)	Excellent (FDA-approved, tunable degradation)	Lactic acid, glycolic acid	Weeks–months (composition dependent)	[98,99,100]
Poloxamer (Pluronic F127)	P407/F127	Synthetic	Poly(ethylene oxide), poly(propylene oxide)	Good–excellent (widely used, non-toxic)	Primarily excreted (minimal degradation)	Hours–days	[101]
Chitosan	-	Natural (modified polysaccharide)	–NH_2_, –OH	Excellent (bioactive, antimicrobial)	Oligosaccharides, glucosamine	Days–weeks (enzyme dependent)	[102,103]

**Table 3:** Preclinical studies using injectable hydrogel and organic nanoparticle platforms for growth factor and cell-mediated liver regeneration. HGF = hepatocyte growth factor, EGF = epidermal growth factor, IPSC = induced pluripotent stem cell, SC = stem cell, MSC = mesenchymal stem cell, PEG = polyethylene glycol, ECM = extracellular matrix, TAA = thioacetamide, LPS = lipopolysaccharide, AATD = alpha-1 antitrypsin deficiency, CCl_4_ = carbon tetrachloride, AST = aspartate aminotransferase, ALT = alanine aminotransferase, ALB = albumin, GOT = glutamic oxaloacetic transaminase, GPT = glutamate pyruvate transaminase, TUNEL = terminal deoxynucleotidyl transferase dUTP nick end labeling, PCNA = proliferating cell nuclear antigen, Ki-67 = antigen Kiel 67.

Regenerative Treatment	Biomaterial Base	Material Dimensions	Location	Liver Damage Model	Regenerative Markers	Key Data	Citation
N/A	Various		Induced liver wound	Surgical	Total blood loss and hemostasis time	Less blood loss and shorter hemostasis time.	[169,215,216,217,218]
Epidermal Growth Factor	Chitosan Microspheres in a gelatin methacryloyl gel	~30 µm	Induced liver wound	Surgical	Blood loss, hemostatic time, AST, ALT, ALB, and inflammatory cell aggregation	~15% PCNA positive cells vs. ~6% in treatments without microspheres	[219]
HGF	Carboxymethyl–hexanoyl chitosan	Nanoparticles	Intrahepatically	TAA	AST, ALT, and total bilirubin	3 days post treatment AST, ALT, TBIL 252.0, 112.0, 1.00 for 50 ng/mL HGF and 500.2, 404.2, 1.35 for 0 ng/mL HGF.	[116]
HGF	Polylactic acid-O-carboxymethylated chitosan	Nanoparticles	Abdominal cavity	Acute liver failure model with hepatocyte transplant	Survival, liver function, and cell proliferation	Mitotic index 10.2% 5 days post-transplant. Ki-67 labeling index 16.8% 7 days post-transplant.	[220]
HGF	Gelatin	60–130 µm	Peritoneal cavity	TAA	Histological score, hydroxyproline, and area of fibrous linkage	2 mg HGF: Histological score of 1.4 and 6.3% fibrosis compared to histological score of 2.8 and 9.3% fibrosis without gelatin encapsulation. 0.4 mg HGF: Histological score of 2.0 and 6.3% fibrosis compared to histological score of 3.2 and 10.9% fibrosis without gelatin encapsulation.	[115]
HGF DNA	Octaarginine peptide-modified nanoparticles	139 ± 4 nm	Tail vein	LPS/D-galactosamine hydrochloride	Serum GOT/GPT and survival	5-day survival 67.5% vs. 33.3% with no treatment.	[221]
HGF/EGF mRNA	Lipid nanoparticles	~80 nm	Retro-orbital injection or tail vein	N/A	Hepatocyte proliferation	61.25 ± 10.66% proliferating cells.	[118]
HGF/EGF mRNA	Lipid nanoparticles	~80 nm	Retro-orbital injection or tail vein	Choline-deficient diet (MASLD)	Steatosis and ALT	Significant increase in serum cholesterol and significant reduction in serum ALT after 2 days following discontinuation of CDE diet and after 8 days following continuation of CDE diet.	[118]
HGF/EGF mRNA	Lipid nanoparticles	~80 nm	Retro-orbital injection or tail vein	Acetaminophen	Necrotic areas, ALT, and TUNEL+ cells (apoptotic)	Significant decrease in ALT 32 h after injury and in TUNEL+ cells 48 h after injury.	[118]
p21, HGF/EGF mRNA	Lipid nanoparticles	~80 nm	Retro-orbital injection	AATD mouse analog or acetaminophen with hepatocyte transplantation	Serum albumin, hepatocyte engraftment, and ALT	Enhanced hepatocyte engraftment, lower ALT, and higher serum albumin.	[24]
HGF and Cultured Hepatocytes	Liver-specific extracellular matrix or type I collagen	Transplanted macroscale gel	Subcutaneously	Partial hepatectomy	Hepatocyte survival in vivo	17.6% survival in Type I Collagen Gel, 27.4% survival in Liver ECM gel.	[117]
Porcine Hepatocytes	Self-assembling peptide nanofiber (SAPNF) solution	Macroscale injected gel	Spleen	90% hepatectomy	Survival, albumin expression, and apoptotic signals	30 day survival rates for SAPNF + Hepatocyte mice was 70%, 50% for collagen + hepatocytes, 40% for just hepatocytes, 0% for mice receiving no transplanted cells. Expression of albumin increased with transplanted mice and apoptotic events decreased in immunofluorescent study.	[190]
Human Hepatocytes	Alginate	350–600 µm	Peritoneal cavity	80% partial hepatectomy	Survival, ammonia, glucose level, regenerative signals	200 h mortality rate of mice reduced from 94% not receiving hepatocytes to ~30% for mice receiving transplanted encapsulated hepatocytes. Ammonia levels significantly reduced in treatment group and Glucose levels significantly increased.	[160]
Rat Hepatocytes	Alginate	~580 µm	Peritoneal cavity	Galactosamine	Survival, ammonia, AST, ALT, bilirubin, creatinine, and prothrombin time	AST, ALT, and bilirubin levels significantly reduced in rat models receiving encapsulated hepatocytes. 3 day survival rates of rats receiving transplanted hepatocytes was 100% compared to 80% control and 50% rats receiving just microbeads.	[161]
HGF and IPSCs	Carboxymethyl–hexanoyl chitosan (CHC)	Nanoparticles	Intrahepatically	TAA	Survival and % necrosis	14-day survival rates 0% with no HGF and ~30% with 50 ng/mL. ~60% reduction in necrotic area with 50 ng/mL compared to no HGF. ~40% necrotic area reduction with HGF loaded in CHC compared to PBS.	[116]
Human Adipose Derived SCs	Heparin and PEG	500 µm	Tail vein	N/A	HGF secretion and stem cell biodistribution	Human HGF levels in rat serum 31 days after injection with cells in a hydrogel was ~2 ng/mL higher than in rats that only had cells implanted. Average fluorescence for rats with hydrogel-encapsulated cells ~6× higher than just implanted cells after 30 days.	[191]
Human Adipose Derived SCs	Decellularized ECM and antioxidant nanozyme-loaded nanofibers	4.0 nm diameter nanofibers	Liver surface	CCl_4_	Serum AST, ALT, Albumin, regions of necrosis and regeneration, and inflammatory markers	AST reduced ~66% compared to control after 1 day. ALT Reduced ~75% compared to control after 1 day. Albumin level slightly increased compared to control. Necrosis reduced ~50% compared to control.	[222]
Human Adipose Derived MSCs	Puramatrix peptide hydrogel	Macroscale injected gel	Intra-mesenterium space	Methionine and choline deficient diet (MASLD) followed by partial hepatectomy	ALT, remnant liver weight/whole liver weight ratio (regeneration rate), PCNA and Ki-67 regenerative markers, TUNEL apoptosis marker	Increased 30 h and 7-day regeneration rate. Increased PCNA+ cells 30 h post-hepatectomy. Decreased TUNEL+ cells and lower serum ALT 9 h following hepatectomy.	[223]
MSC Extracellular Vesicles	PEG	Macroscale injected gel	Peritoneal cavity	TAA	Necrosis, apoptosis, and inflammatory markers	Necroinflammatory markers reduced ~66% compared to mice not receiving EVs. AST and ALT reduced ~50%.	[199]
MSC Extracellular Vesicles	Carboxymethyl chitosan and oxidized hyaluronic acid	87.1 nm	Induced liver wound	70% Hepatectomy	Liver/body weight ratio. Proliferation markers. AST/ALT.	Increased liver/body weight ratio 1 and 3 days postoperatively, but not after 7 days. Reduced AST/ALT compared to hydrogel only but not to EVs without hydrogel. Increase in Ki67 and PCNA positive cells after 24 h.	[224]

## Data Availability

Not applicable. No new data were created or analyzed in this study.
